# Effects of Dietary Inclusion of Dry *Hydrastis canadensis* on Laying Performance, Egg Quality, Serum Biochemical Parameters and Cecal Microbiota in Laying Hens

**DOI:** 10.3390/ani11051381

**Published:** 2021-05-13

**Authors:** Tzuen-Rong J Tzeng, Tzu-Yu Liu, Chiao-Wei Lin, Pei-En Chang, Pei-Xin Liao, Wen-Yuan Yang, Chih-Yuan Cheng, Pei-Chun Liao, Wen-Dee Chiang, Shih-Torng Ding, Yuan-Yu Lin

**Affiliations:** 1 Department of Biological Sciences, Clemson University, Clemson, SC 29634, USA; tzuenrt@clemson.edu; 2 Department of Animal Science and Biotechnology, Tunghai University, Taichung 407, Taiwan; jane890325@gmail.com (T.-Y.L.); jimmy890322@hotmail.com.tw (C.-Y.C.); 3 Department of Animal Science and Technology, National Taiwan University, Taipei 106, Taiwan; linchiaowei1980@icloud.com (C.-W.L.); iop851213@gmail.com (P.-X.L.); r09626024@ntu.edu.tw (W.-Y.Y.); sding@ntu.edu.tw (S.-T.D.); 4 Institute of Biotechnology, National Taiwan University, Taipei 106, Taiwan; r09642004@ntu.edu.tw; 5 Department of Life Science, National Taiwan Normal University, Taipei 116, Taiwan; pcliao@ntnu.edu.tw; 6 Department of Food Science, Tunghai University, Taichung 407, Taiwan; wdc@thu.edu.tw

**Keywords:** gut health, *Hydrastis canadensis*, microbial community, laying hen

## Abstract

**Simple Summary:**

Extensive studies on alternative sources of feed additives to replace antibiotics are required. Plants of the Ranunculaceae family have been used as medicines or dyes. The representative plant is goldenseal (*Hydrastis canadensis*), which has a long history of use in North America, with berberine considered the most effective ingredient in goldenseal. Some natural compounds in *Hydrastis canadensis* function as efflux pump inhibitors and thus may have bactericidal effects against pathogens with antibiotic resistance. However, no conclusive beneficial effects of goldenseal on the livestock industry have been reported. The objective of this study was to examine whether the alternative use of goldenseal roots or leaves has potential health benefits in chickens. Our data demonstrate that *Hydrastis canadensis* can improve the egg quality and modulate the microbiota composition of laying hens.

**Abstract:**

Alternative growth promoters are able to not only effectively replace the traditional use of antibiotics but also provide additional health benefits for livestock and reduce food safety concerns. This study investigated the effects of dry *Hydrastis canadensis* on the laying performance and fecal microbial community of laying hens. Twenty-four Lohmann (LSL, white layer strain) hens were reared from 40 to 48 weeks of age and randomly allotted to four dietary treatments (six birds/treatment). The dietary treatments comprised a basal diet with no treatment as control, a basal diet plus 0.6% powder of dry *Hydrastis canadensis* roots (R) or leaves (L), and a basal diet plus 0.6% powder of a mixture of dry *Hydrastis canadensis* roots and leaves (1:1, LR). No mortality was observed in the whole experimental period. The results indicated that albumen height in the LR group was significantly greater than that in the control group. The diet supplemented with *Hydrastis canadensis* had no significant effects on egg production rate, egg weight, eggshell strength, eggshell thickness, Haugh unit, or yolk height during the whole experimental phase. However, principal coordinate analysis, comparative heat map analysis, and cluster dendrogram analysis of cecal microbiota showed distinct clusters among the groups treated with *Hydrastis canadensis* and the control group. Regarding blood biochemical parameters, serum cholesterol levels were significantly lower in all *Hydrastis canadensis*-treated groups compared with those in the control group. Moreover, serum low-density lipoprotein levels were lower in hens supplemented with the leaf of *Hydrastis canadensis.* The abundances of the phyla Fusobacteria and Kiritimatiellaeota were increased (*p* < 0.05) in laying hens fed with 0.6% *Hydrastis canadensis* leaves, whereas the abundance of the phylum Firmicutes in cecum digesta decreased in response to treatment with *Hydrastis canadensis* roots and leaves. The relative abundance of the *Fusobacterium* genus was higher in the LR group compared with that in the control. On the contrary, we found a different trend in the *Synergistes* genus. The potential influences of these microbiota on the performance of laying hens were discussed. The results demonstrate that *Hydrastis canadensis* can improve the egg albumen height and modulate the cecum digesta microbiota composition of laying hens.

## 1. Introduction

Goldenseal (*Hydrastis canadensis*) belongs to the family Ranunculaceae. It is a small hairy perennial that emerges in early spring (mid-March to early May) and dies back in mid-August to mid-September. The natural range of the plant extends from southern New England and westwards through the extreme southwestern portion of southern Ontario to southern Wisconsin, and southwards to Arkansas and northern Georgia (USDA Plant Profile). The National Toxicology Program is currently investigating the toxicology of goldenseal root powder. A rapid ambient extraction method to assay goldenseal root powder and determine its purity has been developed [1]. The main components of goldenseal are its alkaloids: berberine, hydrastine, and canadine. It also possesses secondary metabolites, such as protoanemonin and glycosides. In addition, berberastine, meconin, chlorogenic acid, phytosterins, resins, albumin, starch, sugars, lignin, and volatile oil (in the root) are other compounds found in goldenseal [2].

Historically, Native Americans have used goldenseal for various health conditions, including skin diseases, ulcers, and gonorrhea. Its roots and rhizomes, which internally are bright yellow in color, have been used as a traditional medicine for the treatment of infection and inflammation and as an immune system booster [3]. More recently, it is known to be taken orally to treat upper respiratory infections and gastrointestinal tract disorders and is commonly found in commercial products in combination with *Echinacea purpurea* [4,5]. Goldenseal and its chemical constituents have been reported to exhibit many antimicrobial properties, including anti-bacterial, anti-fungal [6,7,8], anti-parasitic [9], and anti-viral [10] activities. The anti-bacterial properties could be due to the bactericidal/bacteriostatic [11,12] activities resulting in the direct inactivation of the bacteria. This could also be due to other indirect interventions, such as the disruption of quorum sensing and biofilm formation [13], acting synergistically with the anti-bacterial constituents in goldenseal [14,15], via the inhibition of bacterial drug efflux machineries that confer bacterial multi-drug resistance [16], or through overall enhancement of the host immune system [17]. It has also been demonstrated to be a natural LDL-lowering agent [18], an anti-inflammatory agent [4,19,20], and an anti-cancer agent [21,22].

Canadine is known for its sedative and muscle relaxant properties [23]. Hydrastine is a valuable drug in the treatment of diseases of the skin. Both of them are taken internally into the body or as a topical application.

Two new C-methyl flavonoids, 6,8-di-C-methylluteolin 7-methyl ether and 6-C-methylluteolin 7-methyl ether, have been isolated, and these show activity against oral pathogens, such as *Streptococcus mutans* and *Fusobacterium nucleatum* [24]. Quinic acid feruloyl esters have also been identified, and their activities against *Mycobacterium tuberculosis* have been evaluated but have been shown not to have a significant effect [25].

It is reasonable to conclude that since goldenseal contains a slew of constituents with various antimicrobial properties, efflux pump inhibitors, and immune modulators, we could utilize goldenseal as alternative to antibiotics and as performance enhancer in animal production. To date, a limited number of trials evaluating the use of botanicals as growth promoters have been carried out [26,27,28,29]. Rajaian et al. evaluated the root powder of *Berberis vulgaris* for its potential as a growth promoter in broiler chickens and reported that chickens fed with diets containing 1% root powder from day 1 were significantly heavier than birds in the control group [28]. In addition, they reported that diets containing 2% root powder were less effective and gave the decreased palatability of the feed as the reason for this. Xiao et al. reported that berberine can be included safely in poultry feed up to the highest concentrations tested (i.e., 0.3 g/kg) [29]. However, no conclusive beneficial results, such as improved weight gains or feed conversion ratio, were reported. It is worth noting that these trials only evaluated root powders or pure berberine compounds without the inclusion of the leaf part.

In this study, we evaluated the use of 0.6% root of goldenseal, 0.6% leaf of goldenseal, and 1:1 root/leaf goldenseal preparations and determined the potential health benefits of using such alternative agents in animals. We anticipated that the inclusion of whole plant products should provide additional performance benefits.

## 2. Materials and Methods

### 2.1. Goldenseal Source Materials

Goldenseal plant materials were provided by Sleepy Hollow Farm (628 Sleepy Hollow Road, Powder Springs, GA, USA). The plant materials were produced under the USDA National Organic Program certified production, processing, and standardization system for goldenseal. The composition of the goldenseal materials is shown in Table 1.

### 2.2. Laying Hens and Experimental Design

All experiments were carried out in accordance with approved guidelines. The protocol for animal experiments was approved by the Institutional Animal Care and Use Committee of Tunghai University. Twenty-four Lohmann (LSL, white layer strain) laying hens were reared from 40 to 48 weeks of age and randomly allotted to 4 dietary treatments (6 birds/treatment). The dietary treatments were as follows: (1) a basal diet with no treatment as control, (2) a basal diet plus 0.6% root of *Hydrastis canadensis* (R), (3) a basal diet plus 0.6% leaf of *Hydrastis canadensis* (L), and (4) a basal diet plus 0.3% leaf and 0.3% root of *Hydrastis canadensis* (LR). Chickens were housed in floor pens at 25 °C under a 14 h light/10 h dark cycle. Water and feed were provided ad libitum during the whole experimental period.

The diets were formulated to meet the requirements of laying hens according to breeder recommendations. The compositions and nutrient levels of the diets used in the current study are presented in Table 2.

### 2.3. Proximate Analysis

The percentages of crude protein in the leaves and roots of goldenseal were analyzed using the Kjeldahl method [30]. Briefly, samples of 0.3 g ammonium (II) sulfate hexahydrate, as an analytical standard (Merck, Darmstadt, Germany), were wrapped in individual weighing papers. The samples were digested in digestion flasks with a Kjeldahl tablet (Merck, Darmstadt, Germany) and 10 mL of concentrated sulfuric acid (Sigma-Aldrich Corporation, St Louis, MO, USA), while a digestion flask without samples was regarded as a blank. Distillation was performed using a KjelFlex K-360 distillation unit (Büchi Labortechnik AG, Flawil, Switzerland). After distillation, the ammonia was captured using the KjelFlex K-360 distillation unit in 25 mL of 4% boric acid (Sigma-Aldrich Corporation, MO, USA) with 0.1% bromocresol green (Sigma-Aldrich Corporation, St Louis, MO, USA) and 0.1% methyl red (Sigma-Aldrich Corporation, MO, USA). Then, 0.1 N sulfuric acid (Sigma-Aldrich Corporation, St Louis, MO, USA) was used to titrate the distilled material until the color changed to pink. The nitrogen contents were calculated using the following equation: $Nitrogen\;\left(\%\right)=\frac{\left(V_{\;sample}-V_{\;blank}\right)\times N\times 14.01}{sample\;weight\;\left(g\right)\times 1000\times recovery\;rate\;of\;standard}\times 100\;\%$. *V _sample_*: titrated volume (mL) used in sample, *V _blank_*: titrated volume (mL) used in blank, *N*: normality of H_2_SO_4_. The percentage of crude protein was calculated using the following equation: Crude protein (%)=Nitrogen (%)×6.25. We referred to a procedure by Van Soest et al. [31] to measure the percentage of neutral detergent fiber in the goldenseal leaves and roots. Briefly, to determine the percentage of neutral detergent fiber (NDF), 0.5 g of sample was put in a fiber bag (C. Gerhardt, Königswinter, Germany) and weighed. Then, the samples were soaked in neutral detergent solution (3% sodium lauryl sulfate, 1.86% EDTA, 0.681% sodium tetraborate decahydrate, 0.456% disodium hydrogen phosphate dihydrate, and 1% 2-ethoxyethanol) containing 0.175% sodium sulfite, 1 to 2 drops of decalin, and heat-stable α-amylase (#A3306, Sigma-Aldrich Corporation, St Louis, MO, USA). After boiling for 1 h, the materials within the fiber bags were sequentially washed using hot distilled water and acetone. After evaporation at 105 °C, the residues of the samples were weighed. The percentage of neutral detergent fiber was calculated using the following equation: $Neutral\;detergent\;fiber\;\left(\%\right)=\frac{Weight_{sample\;and\;bag}-Weight_{bag}-Weight_{residue\;}}{Weight_{sample}}\times 100\;\%$. The residues of the samples with the fiber bags were further used to analyze their percentage of acid detergent fiber using the procedure described in a previous study [31]. Briefly, the samples were soaked in acid detergent solution (2% N-cetyl-N, N-trimethylammonium bromide and 2.744% sulfuric acid) with 1 or 2 drops of decalin. After boiling for 1 h, the samples were washed using distilled water and acetone sequentially. The samples were dried at 105 °C and then weighed. The percentage of acid detergent fiber was calculated using the following equation: $Acid\;detergent\;fiber\;\left(\%\right)=\frac{Weight_{sample\;and\;bag}-Weight_{bag}-Weight_{residue\;}}{Weight_{sample}}\times 100\;\%$. The residues of the samples were used to determine the percentage of acid detergent lignin. The samples were soaked in 78% H_2_SO_4_, which was refreshed every 1 h for a total of 4 h. The samples were washed using hot water to be neutral and then washed using acetone. The samples were dried at 105 °C. To obtain ash, the dried samples were incubated at 600 °C for 8 h. The percentage of acid detergent lignin was calculated using the following equation: $Acid\;detergent\;lignin\;\left(\%\right)=\frac{Weight_{sample\;and\;bag}-Weight_{bag}-Weight_{ash}}{Weight_{sample}}\times 100\;\%$.

### 2.4. DNA Extraction and Microbiota Analysis

At the end of the experiment (48-week-old laying hens), cecal contents were collected for bacterial DNA extraction. For microbiota analysis, bacterial DNA was extracted from 200 mg of cecal contents from pooled samples (two cecal contents pooled together and sample sizes of 3 for each group). The DNA was extracted using a commercial kit (QIAamp Fast DNA Stool Mini Kit; Qiagen, Hilden, Germany) following the instructions of the manufacturer. The quality and quantity of DNA was evaluated on a SimpliNano^TM^ spectrophotometer (SimpliNano, 29,061,711). DNA samples were stored at −20 °C until further processing. DNA amplicons from individual samples were amplified with specific primers for the V3 to V4 regions, and libraries were generated by the TruSeq Nano DNA Library Prep Kit (Illumina, San Diego, CA, USA). The libraries were further sequenced on an Illumina MiSeq platform. The 300 bp paired-end raw reads derived from the 16S ribosomal amplicon sequencing were assembled and clustered into operational taxonomic units (OTUs) at 97% identity. A Venn diagram was also used to compare the similarities and differences between the 4 groups.

### 2.5. Laying Performance and Egg Quality

Laying performance was evaluated during the whole experimental trial. Egg production and mortality were recorded daily. All eggs produced at the end of the experiment were collected in 2 consecutive days, and egg quality parameters (egg weight, albumen height, Haugh unit, eggshell thickness, and yolk color score) were measured. Eggshell thickness was measured with an eggshell thickness gauge (Model-ID-C1012EXBS, Mitutoyo Co., Kawasaki, Japan). Eggshell strength was measured with an Egg Force Reader (Model-HI-8116, Hung Ta Co., Taichung, Taiwan). Albumen height and yolk height were determined with an egg analyzer (Model-NFN381, Fujihira, Tokyo, Japan). Yolk color score was determined using a yolk color chart (Robotmation Co., Tokyo, Japan).

### 2.6. Serum Biochemical Parameters

At the end of the experimental trial, blood samples were collected from the wing vein. The blood samples were centrifuged at 2500× *g* for 15 min at 4 °C to collect serum, and the serum samples were stored at −80 °C. The serum samples were diluted to appropriate concentrations with phosphate-buffered saline, and levels of glucose (GLU, #3150, Fujifilm, Tokyo, Japan), triacylglycerol (TG, #1650, Fujifilm, Tokyo, Japan), total cholesterol (CHOL, #1450, Fujifilm, Japan), high-density lipoprotein (HDL, #2650, Fujifilm, Tokyo, Japan), low-density lipoprotein (LDL, #LDL-C, Denka Seiken, Tokyo, Japan), aspartate transaminase (AST, #3150, Fujifilm, Tokyo, Japan), alanine transaminase (ALT, #3250, Fujifilm, Tokyo, Japan), and creatine kinase (CK, #3350, Fujifilm, Japan) were measured by a biochemistry automatic analyzer (Fuji Dri-Chem NX 500i, Fujifilm Co., Tokyo, Japan).

### 2.7. Statistical Analysis

Statistical analyses were performed using GraphPad software (version 5 for Windows). The collected data were tested by means of one-way ANOVA, and the mean differences were compared using Tukey’s comparison test. Significance was declared at *p* ≤ 0.05. Alpha diversity was indicative of the species complexity within individual samples based on different criteria outputs from the QIIME pipeline [32]. Beta diversity analysis was used to evaluate the differences among samples in terms of species complexity [33]. Principal coordinate analysis (PCoA) was performed to acquire principal coordinates for visualization of sophisticated and multidimensional data [34]. PCoA was conducted by using the FactoMineR package and ggplot2 package in R software (v.2.15.3) 

## 3. Results

### 3.1. Effect of Hydrastis canadensis on Laying Performance, Egg Quality, and Blood Biochemical Parameters of Laying Hens

The effects of dietary supplementation with *Hydrastis canadensis* on laying performance and egg quality are shown in Table 3. No significant change in egg production rate was observed among groups during the entire experimental period, and the production rate of each group was maintained at above 90%. The diet supplemented with *Hydrastis canadensis* had no significant effects on egg weight, eggshell strength, eggshell thickness, Haugh unit, or yolk height during the whole experimental phase (*p* > 0.05) but had significant effects on yolk color score and albumen height. Compared with the control group, albumen height in the LR group significantly increased. Interestingly, the yolk color score in the L group significantly increased compared with that in the R group. No mortality was observed in the whole feeding period.

The effect of *Hydrastis canadensis* on the blood biochemicals of laying hens is presented in Table 4. The serum CHOL was significantly lower in all the treated groups compared with the control group. Relative to the control, serum LDL level was lower in hens that received the diet supplemented with *Hydrastis canadensis* leaf. Moreover, serum CK was lower in the R group. It should be noted that there was no significant difference in serum GLU, HDL, TG, AST, or ALT when compared with the control group.

### 3.2. Effect of Hydrastis canadensis on Cecal Digesta Microbiota

**Figure 1:**
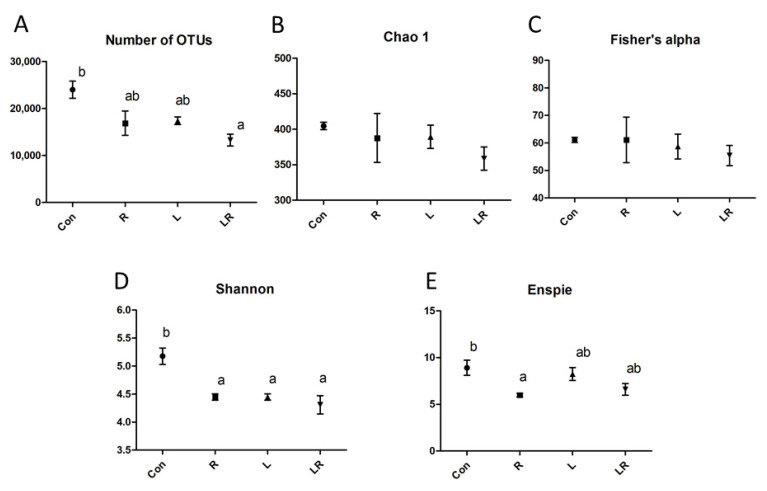
Sample information and microbial diversity in the cecum of laying hens. (**A**) Number of OTUs, (**B**) Chao 1, (**C**) Fisher’s alpha, (**D**) Shannon, (**E**) Enspie. Con, basal diet; R, control plus 0.6% *Hydrastis canadensis* root; L, control plus 0.6% *Hydrastis canadensis* leaf; LR, control plus 0.6% *Hydrastis canadensis* root and leaf. Data are expressed as mean ± SEM. Symbols indicate statistical significance. Different letters indicate *p*-value ≦ 0.05.

**Figure 2:**
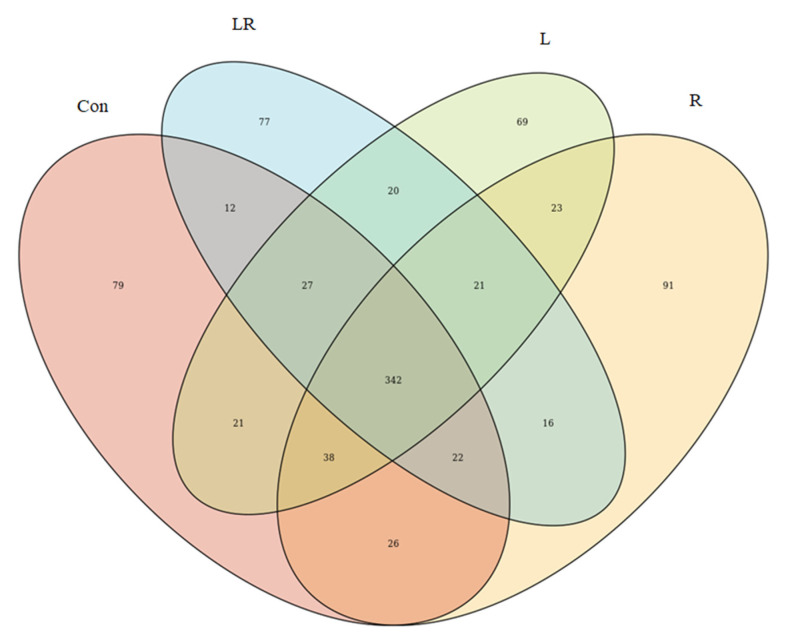
Venn diagram of the operational taxonomic unit distribution of the cecum microbial community. Each ellipse represents one group. The value of each region represents the number of OTUs corresponding to the region. Con, basal diet; R, control plus 0.6% *Hydrastis canadensis* root; L, control plus 0.6% *Hydrastis canadensis* leaf; LR, control plus 0.6% *Hydrastis canadensis* root and leaf.

**Figure 3:**
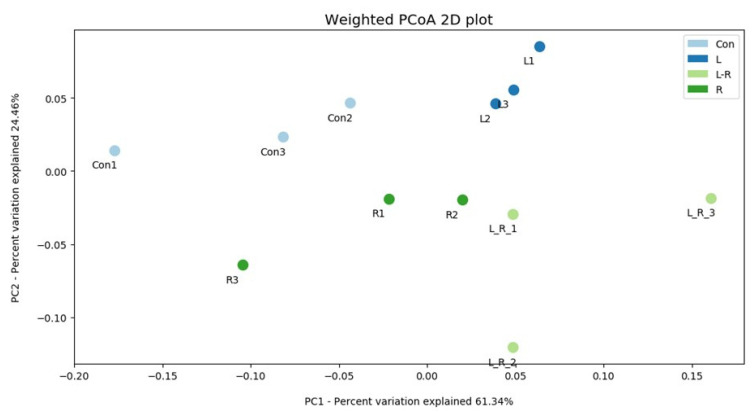
Advanced analysis of bacterial communities in cecum contents. PCoA of weighted UniFrac distance in the cecum contents from Con, R, L, and LR.

**Figure 4:**
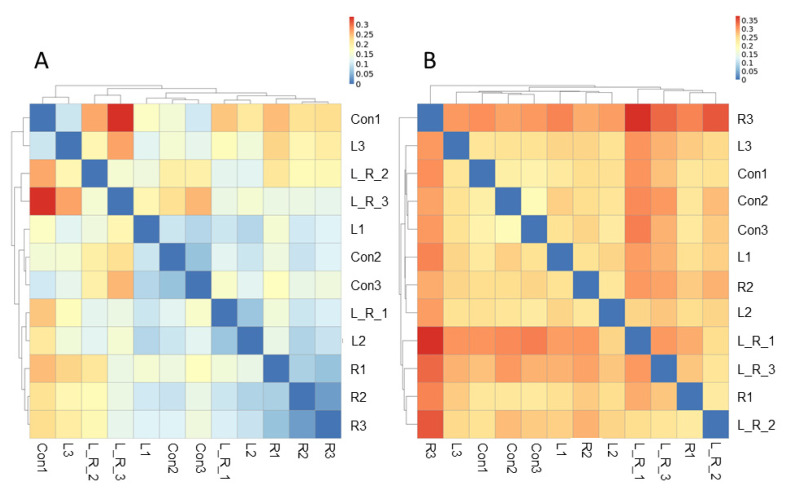
Comparative heat map analysis of the cecum contents. (**A**) The beta diversity index of the cecum content from the basal diet (Con), control plus 0.6% *Hydrastis canadensis* root (R), control plus 0.6% *Hydrastis canadensis* leaf (L), control plus 0.6% *Hydrastis canadensis* root and leaf (LR) on weighted UniFrac metrics. (**B**) The beta diversity index of the cecum content from Con, R, L, and LR on unweighted UniFrac metrics.

The effect of *Hydrastis canadensis* on cecal digesta microbiota of laying hens is presented in Figure 1. After trimming of raw tags, the number of OTUs from the cecum contents of laying hens was approximately 20,000. Chao 1 and Fisher’s alpha analysis showed that species richness was not changed by *Hydrastis canadensis* treatment. However, Shannon and Enspie analysis indicated that species evenness was lower in the groups treated with *Hydrastis canadensis* (Figure 1). A Venn diagram showed a large amount of overlap among the groups (342 OTUs). In total, 79, 69, 91, and 77 unique OTUs were found in the Con, R, L, and LR groups, respectively. Twenty-six OTUs were discovered in both the control group and the group treated with 0.6% *Hydrastis canadensis* root. Twenty-one unique OTUs were discovered in both the control group and the group treated with 0.6% *Hydrastis canadensis* leaf (Figure 2). Data from the principal coordinate analysis based on a weighted UniFrac metric indicated that the microbiota of cecal cecum were clearly divided into different communities among the groups (PC1, 61.34%; PC2, 24.46%; Figure 3). UniFrac is a distance metric used for comparing biological communities using phylogenetic information and indicates beta diversity. The results of beta diversity analysis based on weighted and unweighted UniFrac metrics were consistent with the principal coordinate analysis, which indicated that the microbiota of the fecal samples were differentiated (Figure 4A,B). A cluster dendrogram also showed that the treatment of *Hydrastis canadensis* caused different clusters in microbiota (Supplemental Figure S1).

### 3.3. Effect of Hydrastis canadensis on Bacterial Taxonomic Composition of Cecal Digesta

**Figure 5:**
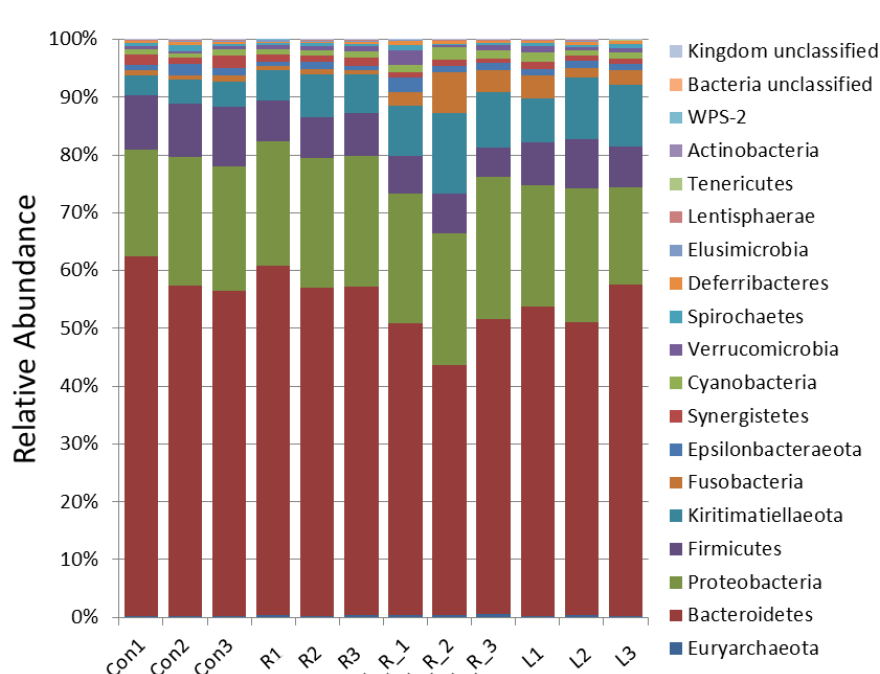
Bacterial taxonomic composition of cecum contents. The composition of the microbiome from cecum contents at the genus level. Values are normalized by Z-score.

The effects of *Hydrastis canadensis* on the bacterial taxonomy composition in the intestinal contents of laying hens are presented in Figure 5. In addition, relative abundances greater than 1% with the phylum level and genus level are further shown in Figures S2 and S3, respectively. Compared with the control group, there were higher abundances of the phyla Fusobacteria and Kiritimatiellaeota in the group treated with 0.6% *Hydrastis canadensis* leaf. No significant changes were observed in the phyla Proteobacteria and Cyanobacteria among the groups. The relative abundance of the phylum Firmicutes in cecum contents was lower in the laying hens of all the treatment groups than those of the control group. Regarding the abundance of the phylum Bacteroidetes, no significant difference was found in *Hydrastis canadensis* leaf or *Hydrastis canadensis* root treatment. However, a combination of *Hydrastis canadensis* leaf and root had a synergistic effect on the phylum Bacteroidetes. At the genus level, there was no difference between the groups in the *Rikenellaceae RC9* genus, *Parasutterella* genus, *Bacteroides* genus, and *Helicobacter* genus. The relative abundance of the *Fusobacterium* genus was higher in the *Hydrastis canadensis* leaf and root group compared with the control. On the contrary, we found a different trend in the *Synergistes* genus.

## 4. Discussion and Conclusions

*Hydrastis canadensis* is a natural plant with a long history of use, and berberine is considered its most effective ingredient [20]. It has been confirmed that *Hydrastis canadensis* can ameliorate several pathological statuses and has anti-inflammation [35], anti-cancer [36], anti-diabetic [37], anti-obesity, and anti-hyperlipidemia properties [38,39], in addition to offering myocardial protection [40]. Our results indicate that laying hens fed with diets containing goldenseal root, leaf, or a mixture of leaf and root (1:1) showed no mortality throughout the experiments. Moreover, a previous study reported that broiler chickens fed with berberine showed no significant gastrointestinal and liver lesions [41]. These indicate that *Hydrastis canadensis* and its most effective ingredient, berberine, may be safe to be used as a feed supplement. However, the effects of *Hydrastis canadensis* on egg quality have not been investigated. In the current research, we found that supplementation with *Hydrastis canadensis* leaf (L) in diets significantly increased the yolk color score of eggs. Additionally, supplementation with *Hydrastis canadensis* leaf and root (LR) significantly increased the albumen height produced by laying hens. These results suggest that supplementation with *Hydrastis canadensis* in diets may improve the quality of eggs and may have the potential to be utilized in the egg industry.

Previous research has suggested that the intestinal microbiota may mediate the improvement of specific diseases [20]. In fact, microorganisms may contribute to the lowering of hyperlipidemia by berberine [42,43]. Regarding livestock animals, limited studies on berberine have been conducted. A previous study demonstrated that supplementation with berberine in lipopolysaccharide-challenged broilers mitigated growth performance impairment, oxidative stress, and inflammatory response [44]. In laying hens, however, little is known about the role of *Hydrastis canadensis* (berberine is the main component) in the regulation of the microbial community. In this study, we demonstrated that principal coordinate analysis and the beta diversity index showed distinct clusters among the groups treated with *Hydrastis canadensis* leaf and root. The abundances of the phyla Bacteroidetes and Firmicutes were lower in the cecum contents of the group treated with the combination of *Hydrastis canadensis* leaf and root. The abundance of the *Fusobacterium* genus was higher in the cecum contents of the group treated with the combination of *Hydrastis canadensis* leaf and root. On the contrary, the abundance of the *Synergistes* genus was lower in the cecum contents of the group treated with the combination of *Hydrastis canadensis* leaf and root.

Moreover, the supplementation with *Hydrastis canadensis* leaf (L), root (R), or a mixture of leaf and root (LR) all reduced the concentrations of serum total cholesterol significantly (*p* < 0.01) in laying hens. This suggests that supplementation with *Hydrastis canadensis* in diets shows hypolipidemic effects in laying hens. Consistent with a previous study, the ethanol extract of *Hydrastis canadensis* and berberine, an effective ingredient in *Hydrastis canadensis*, can reduce the levels of plasma cholesterol in hamsters fed with a high-cholesterol diet [18]. These results suggest that *Hydrastis canadensis* has the potential to prevent or cure diseases attributed to hyperlipidemia in chickens, such as fatty liver diseases [45]. However, the mechanisms by which *Hydrastis canadensis* improves egg quality and lowers the concentrations of total cholesterol in serum are yet to be elucidated. Our results also show that the leaves (L), the roots (R), and the mixture of leaves and roots (LR) of *Hydrastis canadensis* decreased the abundance of the Firmicutes phylum in cecal microbiota. In a clinical study, the Firmicutes phylum was shown to be negatively correlated with levels of low-density lipoprotein cholesterol (LDL-C) in individuals treated with rosuvastatin, a cholesterol-lowering drug [46]. In our results, decreases in plasma cholesterol in laying hens supplemented with *Hydrastis canadensis* were also accompanied by decreases in the Firmicutes phylum in the cecal microbiota. These suggest that the Firmicutes phylum might contribute to the lowering of cholesterol; however, this still needs evidence to be proved.

In the gut, *Fusobacterium* contributes to the production of butyrate and the metabolism of bile acids [47,48,49,50]. Short-chain fatty acids are the metabolites of various nutrients fermented by particular microorganisms in the gut [51]. Previous research has shown that butyrate can reduce blood cholesterol by inhibition of cholesterol absorption in the gut [52,53,54]. Moreover, an in vitro study showed that butyrate inhibits the secretion of cholesterol in Caco-2 cells, a human intestinal cell line [55]. It has been proposed that butyrate might inhibit cholesterol transportation from the colon into circulation to lower blood cholesterol [55]. The increase in *Fusobacterium* in the laying hens fed with diets containing leaves (L) or a mixture of leaves and roots (LR) was associated with a decrease in blood total cholesterol. Therefore, the decrease in total cholesterol in the blood caused by *Hydrastis canadensis* might also be related to the increased *Fusobacterium* in the gut.

Previous research has shown that the function of berberine in reducing obesity or ameliorating hyperlipidemia is almost certain in mammals [42,43]. After db/db mice were fed with 136.5 mg/kg (body weight) of berberine for 11 weeks, short-chain fatty acids were increased in the feces, and the Firmicutes-to-Bacteroidetes ratio, which was found to be increased in the gut microbiota of humans with obesity [56], was also modulated by treatment. In addition, this study observed an increase in the proportions of *Butyricimonas*, *Lactobacillus*, *Coprococcus*, *Ruminococcus*, and *Akkermansia*, while *Prevotella* and *Proteus* were decreased [43]. The modulating function was also found in a rat study. The richness and diversity of the gut microbiota decreased in rats when treated with 150 mg of berberine per body weight for 6 weeks. In addition, the abundances of *Fusobacteria* and *Proteobacteria* increased, in addition to the abundances of *Firmicutes* and *Actinobacteria*. At the genus level, those increased by berberine treatment under hyperlipidemic status include *Fusobacterium*, *Anaerostipes, Bacteroides*, and *Phascolarctobacterium*; on the contrary, those decreased include *Roseburia*, *Allobaculum*, *Oscillibacter*, *Faecalibacterium*, *Prevotella*, *Desulfovibrio*, *Coprococcus*, *Collinsella*, and *Blautia*. The results of the aforementioned study were consistent with our current data regarding laying hens.

In summary, no adverse effects in egg quality were observed among the groups. *Hydrastis canadensis* does not provide additional performance benefits to laying hens according to the results of the study. However, it should be noted that supplementation with *Hydrastis canadensis* in the diet may modulate the cecum microbiota composition of laying hens. More interestingly, *Hydrastis canadensis* can ameliorate hyperlipidemic status in laying hens by lowering CHOL and LDL levels.

## Figures and Tables

**Table 1:** Composition of goldenseal materials.

Ingredient	Root (R)	Leave (L)
Dry matter (DM, %)	96.40	96.81
Crude protein (% DM)	13.36	9.72
Neutral detergent fiber (% DM)	32.67	29.96
Acid detergent fiber (% DM)	29.20	27.47
Acid detergent lignin (% DM)	6.03	5.62
Ether extract (% DM)	0.94	1.98
Ash (% DM)	4.53	11.59
Berberine (%)	0.38	0.11

**Table 2:** Compositions of basal and experimental diets.

Ingredient	Composition (%)			
	Basal Diet	R Diet	L Diet	LR Diet
Corn	52.20	51.89	51.89	51.89
Soybean meal (CP 47%)	30.30	30.12	30.12	30.12
CaCO_3_	11.10	11.03	11.03	11.03
monoCaP	2.20	2.19	2.19	2.19
Soybean oil	3.00	2.98	2.98	2.98
DL-Met	0.30	0.30	0.30	0.30
Salt	0.30	0.30	0.30	0.30
Vitamin premix	0.05	0.05	0.05	0.05
Mineral premix	0.05	0.05	0.05	0.05
NaHCO_3_	0.50	0.50	0.50	0.50
Calculated composition (%)				
5.38 kcal/kg	2953.17	2954.36	2954.36	2954.36
Crude protein (%)	18.05	18.02	18.00	18.01
Crude fat (%)	5.38	5.35	5.35	5.35
Calcium (%)	4.72	4.69	4.69	4.69
Available phosphorus (%)	0.57	0.57	0.57	0.57
Methionine + cysteine (%)	0.93	0.92	0.92	0.92
Dry matter	90.39	90.43	90.43	90.43
Crude protein	17.08	16.98	16.98	16.98
Total fatty acid	5.03	5.00	5.00	5.00

Supplied per kg diet: vitamin A, 15,000 IU; vitamin D_3_, 1200 IU; vitamin E, 45 IU; vitamin K_3_, 3.0 mg; vitamin B_1_, 3.0 mg; biotin, 0.2 mg; folacin, 2.0 mg; Fe, 60 mg; Mn, 40 mg; Zn, 50 mg; Cu, 5 mg; I, 0.05 mg; Co, 0.05 mg.

**Table 3:** Effect of *Hydrastis canadensis* on laying performance and egg quality in laying hens.

	Egg Production	Egg Weight	Eggshell Strength	Eggshell Thickness	Yolk Color Score	Haugh Unit	Albumen Height	Yolk Height
	%	g	N	mm	-	-	mm	mm
Control	91.03	66.34	54.63	0.37	6.33 ^ab^	82.52	6.84 ^a^	18.54
R	90.05	65.05	52.76	0.39	5.83 ^a^	84.03	7.33 ^ab^	18.70
L	91.94	64.21	54.11	0.40	7.20 ^b^	85.87	7.39 ^ab^	19.41
LR	90.44	64.77	51.93	0.37	6.50 ^ab^	84.90	8.27 ^b^	18.92
SEM	1.25	0.71	2.71	0.01	0.27	2.19	0.34	0.39
*p*-Value	0.74	0.25	0.88	0.22	0.05	0.77	0.05	0.49

Each mean represents six replicates. Abbreviations: control (basal diet); R, control plus 0.6% *Hydrastis canadensis* root; L, control plus 0.6% *Hydrastis canadensis* leaf; LR, control plus 0.6% *Hydrastis canadensis* root and leaf; SEM = standard error mean (*n* = 6). Different letters indicate *p*-value ≦ 0.05.

**Table 4:** Effect of *Hydrastis canadensis* on blood parameters in laying hens.

	GLU	TG	CHOL	HDL	LDL	AST	ALT	CK
	mg/dL	mg/dL	mg/dL	mg/dL	mg/dL	U/L	U/L	U/L
Control	275.40	2121.60	147.20 ^a^	25.40	84.40 ^a^	231.80	10.20	2077.40 ^a^
R	232.83	1289.83	58.33 ^b^	11.00	76.17 ^ab^	176.33	10.00	974.67 ^b^
L	277.33	1958.33	70.17 ^b^	23.33	74.83 ^b^	205.80	10.17	1246.50 ^ab^
LR	274.33	1914.5	52.00 ^b^	26.50	78.00 ^ab^	185.60	10.00	1189.20 ^ab^
SEM	23.48	218.58	14.09	5.97	2.03	30.02	0.09	246.22
*p*-Value	0.48	0.10	<0.01	0.31	0.03	0.60	0.58	0.03

Each mean represents six replicates. Abbreviations: GLU, glucose; TG, triglyceride; CHOL, cholesterol; HDL, high-density lipoprotein; LDL, low-density lipoprotein; AST, aspartate transaminase; ALT, alanine transaminase; CK, creatine kinase. Control (basal diet); R, control plus 0.6% *Hydrastis canadensis* root; L, control plus 0.6% *Hydrastis canadensis* leaf; LR, control plus 0.6% *Hydrastis canadensis* root and leaf; SEM = standard error mean (*n* = 6). Different letters indicate *p*-value ≦ 0.05.

## Data Availability

The data presented in this study are available on request from the corresponding author.

## Supplementary Materials

The following are available online at https://www.mdpi.com/article/10.3390/ani11051381/s1, Figure S1: Cluster dendrogram analysis; Figure S2: Relative propositions that the bacterial communities in cecum contents were greater than 1% (phylum level); Figure S3: Relative propositions that the bacterial communities in cecum contents were greater than 1% (genus level).
