# Supplementary figures and images for: Effects of Dietary Inclusion of Dry Hydrastis canadensis on Laying Performance, Egg Quality, Serum Biochemical Parameters and Cecal Microbiota in Laying Hens
Source: Animals (Basel). 2021 May 13;11(5):1381. doi: 10.3390/ani11051381 (PMC8152295; doi:10.3390/ani11051381)

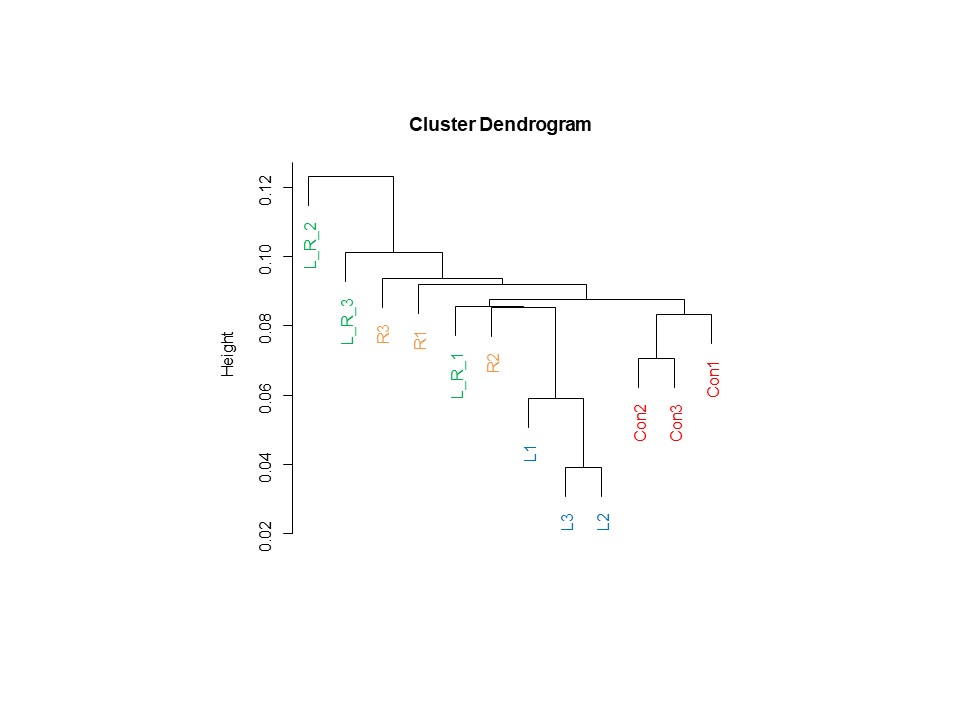

Supplement: Supplementary file 1 [file animals-11-01381-s001.zip › Supplemental Figure 1.jpg]

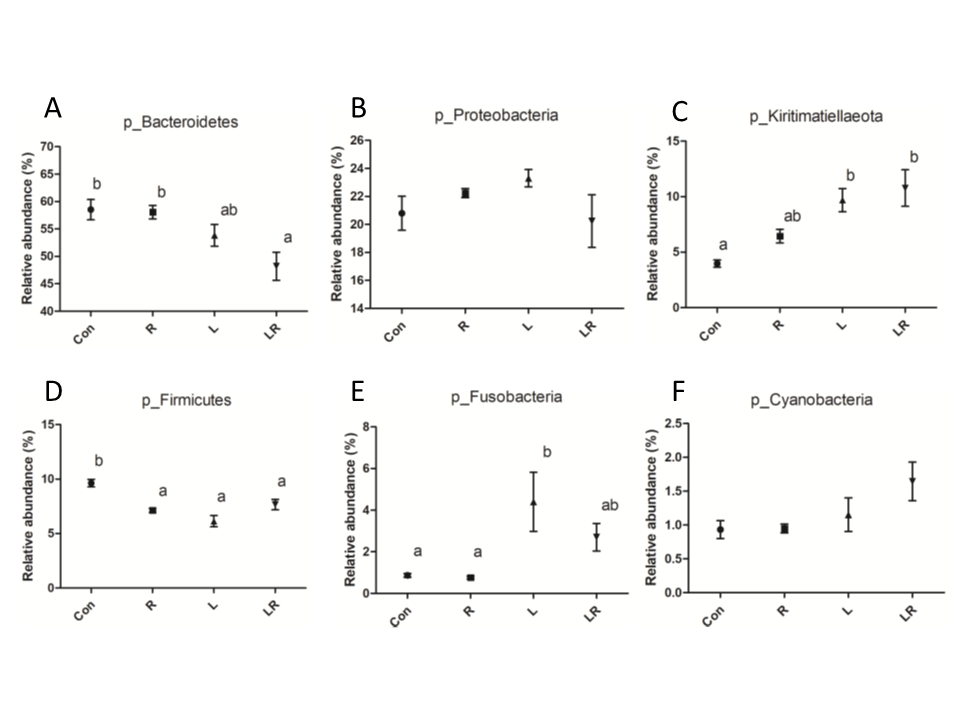

Supplement: Supplementary file 1 [file animals-11-01381-s001.zip › Supplemental Figure 2.tif]

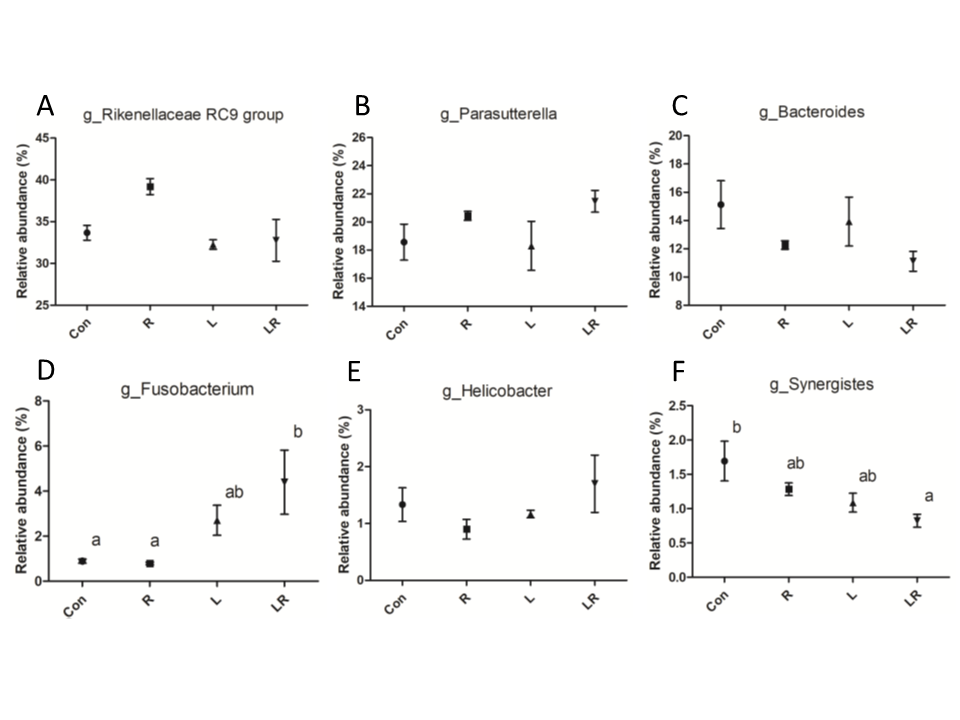

Supplement: Supplementary file 1 [file animals-11-01381-s001.zip › Supplemental Figure 3.tif]
